# Topical Timolol Vs. Oral Propranolol for the Treatment of Superficial Infantile Hemangiomas

**DOI:** 10.3389/fonc.2018.00605

**Published:** 2018-12-18

**Authors:** Hai Wei Wu, Xuan Wang, Ling Zhang, Jia Wei Zheng, Chao Liu, Yan An Wang

**Affiliations:** ^1^Department of Oral and Maxillofacial Surgery, Shanghai Ninth People's Hospital, College of Stomatology Shanghai Jiao Tong University School of Medicine, Shanghai, China; ^2^Department of Oral and Maxillofacial Surgery Shandong Provincial Hospital Affiliated to Shandong University, Jinan, China

**Keywords:** infantile hemangioma, topical timolol, oral propranolol, superficial type, side effects

## Abstract

**Objective:** Infantile hemangiomas (IHs) are the most common vascular tumors of infancy. Oral propranolol has achieved great success in treating IHs since 2008. To minimize the systemic side events caused by oral administration of propranolol, topical timolol started to be applied in the treatment of IHs, especially for superficial lesions.

**Methods:** We treated 724 children with superficial IHs using oral propranolol or topical timolol, and investigated the efficacy and safety of the two treatment patterns.

**Results:** Both oral propranolol and topical timolol achieved a satisfactory therapeutic outcome, with an effective response rate of 97 and 96.4%, respectively. No significant differences in visual analog scale (VAS) improvement between the two groups were observed. Occurrence rate of systemic adverse events for patients treated with oral propranolol (3.9%) was significantly higher than that for patients treated with topical timolol (0%). Clinical response was not associated with gender, duration of treatment, lesion location, lesion size, gestational age, and progesterone use during pregnancy, but closely associated with age at treatment initiation, which indicated that younger age at treatment initiation predicted for a better regression rate.

**Conclusions:** We recommend that topical timolol instead of oral propranolol could be the first-line therapy for superficial IHs because of its good efficacy and improved safety.

## Introduction

Infantile hemangiomas (IHs) are the most common vascular tumors of infancy (1). The cause of hemangioma is still unknown, but it is closely associated with the disorder of angiogenesis and vasculogenesis (2). Owing to the characteristic growth pattern of IHs with rapid proliferation and followed involution, conservative therapy strategies based on observation without early interference were prevalent over several decades (3). However, observational treatment failed to achieve satisfactory therapeutic effects because of the slow rate of tumor regression and permanent residuals leading to cosmetic problems (4). Thus, early and active intervention has become the first choice for proliferating IHs.

In 2008, Léauté-Labrèze et al. (5) reported their results of successfully treating IHs with oral propranolol. Since then, propranolol has become the first-line drug for IHs, but its molecular mechanisms are not well-elucidated (6). Furthermore, several systemic drug adverse events (AEs) have been observed in certain patients after oral propranolol (7). To minimize potential side effects caused by systemic use of propranolol, topical timolol started to be applied in the treatment of IHs, especially for superficial lesions (8–11). In our previous study, it has been confirmed that topical application of 0.5% timolol maleate hydrogel is safe and effective for superficial IHs (12). However, it is still controversial whether topical timolol is superior to oral propranolol in the treatment of superficial IHs, because few clinical studies have focused on comparing the safety and efficacy of topical timolol with that of oral propranolol. In the present study, we investigated patients with superficial IHs receiving either topical timolol or oral propranolol, and aimed to compare the efficacy and safety of two treatment patterns in a large case series.

## Materials and Methods

### Study Design

The study protocol was carried out in accordance with Declaration of Helsinki, and informed consents were obtained from all guardians of the patients. The study was approved by the Institute Review Board of Shanghai Ninth People's Hospital, and conducted between January 2010 and January 2017 at Department of Oral and Maxillofacial Surgery, Shanghai Ninth People's Hospital. Consecutive patients diagnosed as superficial IHs were collected in the present study. The exclusion criteria included a history of previous medication, contraindications of β-blockers, other IH lesions including ulcerated, mucosal, mixed, or deep IHs.

### Treatment Regimen and Outcome Assessment

Before the initiation of treatment, all patients received a thorough physical examination. Clinical features and images of superficial lesions were recorded prior to the treatment. The study samples were consisted of both propranolol-treated lesions and timolol-treated lesions. For oral prorpanolol treatment, patients were given oral propranolol (Jiangsu Yabang Aipusen Pharmaceutical Industry Limited Company, China) at a dose of 2.0 mg/kg per day. Propranolol was divided into 2 doses and taken within half hours after meals. For topical timolol treatment, timolol maleate 0.5% hydrogel was applied three times a day. The hydrogel was gently rubbed as a thin layer onto the whole surface of IH. Cardiovascular examination (including heart rate and blood pressure) was demanded before and after the first application of propranolol or timolol. The treatment continued until objective goals were obtained or no further improvement was achieved.

To record systemic or local AEs, all the patients' guardians were given a questionnaire survey that outlined all potential AEs, including erythema, oedema, crusting, erosion, ulceration, local infections, asthma, bradycardia, hypotension, hypoglycaemia, peripheral vasoconstriction, gastrointestinal disturbances, behavioral changes, sleep disturbances, and diarrhea (13). The data were collected after the treatment for evaluating the safety of two treatments.

Therapeutic responses were defined as blanching and softening of the lesions after treatment initiation. The therapeutic efficacy was mainly evaluated by using visual analog scale (VAS) (14). All clinical photographs of IHs before and after treatment were checked by another three independent physicians who were blinded to the treatment pattern. The VAS score was determined by the change in cosmetic appearance, which ranges from −100 (representing a doubling in the size and extent of the IH) to 100 (representing complete resolution) (12). Therapeutic responses were graded as follows: excellent (VAS score ranging from 90 to 100), good (VAS score ranging from 51-90), fair (VAS score ranging from 1-50) and poor (VAS score ranging from −100 to 0).

### Statistical Methods

Software package SPSS (version 16.0; SPSS, Chicago, IL) was used to analyze the date. Descriptive data were expressed as numbers, percentages, or means ± standard deviations. Mann-Whitney *U-*test was used to compare the clinical responses with different clinical variables, and Fisher's exact test was used to evaluate the differences in the efficacy and safety between two groups. *P* < 0.05 were considered as significant.

## Results

### Clinical Features of Patients

The clinical characteristics of patients included were listed in Table 1. The mean age at initiation of the treatment was 5.8 months. The ratio of female to male was 2.79:1, and 20.9% (151/724) of patients were born prematurely. 11.5% (83/724) of patients had a history of progesterone use. 58.1% (421/724) of lesions were located in the head and neck region. Tumor size ranged from 0.5 to 21.2 cm^2^, with a mean size of 4.42 cm^2^. The mean duration of treatment was 6.7 months, and the mean follow-up time was 6.4 months.

**Table 1:** Clinical characteristics of patients included.

**Characteristic**	**Value**
Age (months)	5.8
Gender	
Male	191
Female	533
Lesion location	
Head and neck	421
Extremities	165
Trunk	138
Lesion size (cm^2^)	4.42
Gestational age	
Tern born (≧37 weeks)	573
Born prematurely (< 37 weeks)	151
Progesterone use during pregnancy	
Yes	83
No	641
Duration of treatment (months)	6.7
Follow-up period (months)	6.4

As shown in Table 2, 724 patients were included in the study, including 362 patients treated with oral propranolol and 362 patients treated with topical timolol. No significant differences in age, gender, lesion location, gestational age, history of progesterone use, treatment duration, and follow-up time were observed in two groups. For patients treated with oral propanolol, the mean age at treatment initiation was 6.1 months. A female predominance with a ratio of 2.9–1 was noted. The primary locations included head and neck region (221), torso (81), and extremities (60). There were 73 patients (20.1%) born prematurely, with 38 patients' mothers (10.5%) having a history of progesterone use during pregnancy. The mean duration of oral propranolol treatment was 6.0 months, and the mean follow-up time was 6.2 months. For patients treated with topical timolol, the mean age at treatment initiation was 5.4 months. A female predominance (2.7:1) was also noted. The lesion were located in the head and neck region (200), torso (84), and extremities (78). There were 78 patients (21.5%) born prematurely, with 45 patients' mothers (12.4%) having a history of progesterone use during pregnancy. The mean duration of topical timolol treatment was 7.3 months, and the mean follow-up time was 6.5 months.

**Table 2:** Clinical characteristics of patients treated with propranolol or timolo.

	**Propranolol**	**Timolol**	***P-*value**
	**group**	**group**	
Age (months)	6.1	5.4	0.62
Gender			0.74
Male	93	98	
Female	269	264	
Lesion location			0.18
Head and neck	221	200	
Extremities	81	84	
Trunk	60	78	
Lesion size			0.21
0–5 cm^2^	235	251	
≧5 cm^2^	127	111	
Gestational age			0.71
Tern born (≧37 weeks)	289	284	
Born prematurely (< 37 weeks)	73	78	
Progesterone use during pregnancy			0.48
Yes	38	45	
No	324	317	
Duration of treatment (months)	6.0	7.3	0.23
Follow-up time (months)	6.2	6.5	0.27

### Therapeutic Outcomes

**Figure 1:**
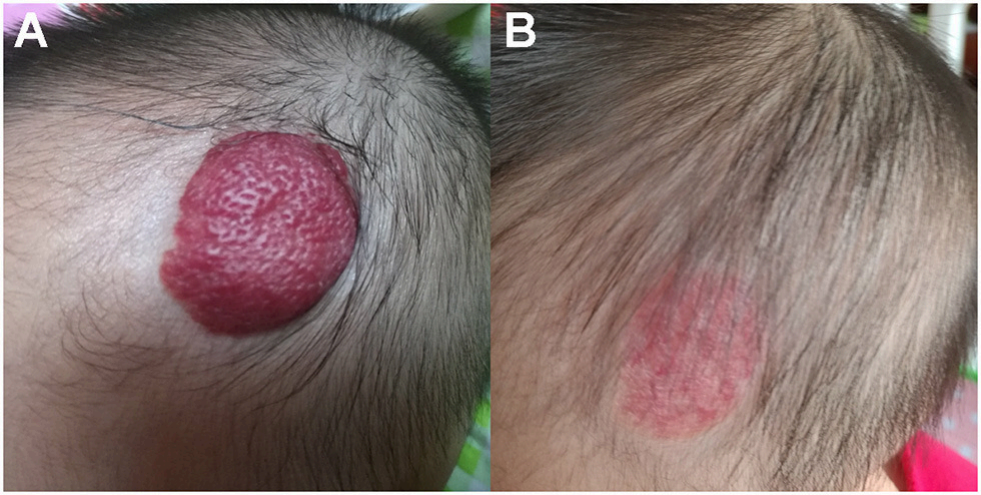
Therapeutic response of superficial IHs in the scalp treated with oral propranolol. **(A)** Before starting oral propranolol treatment; **(B)** After 6 months of oral propranolol treatment. Obvious discoloration and regression in size were noted. Written informed consent has been obtained from the parents of the child, and permission was granted for publication of these images.

**Figure 2:**
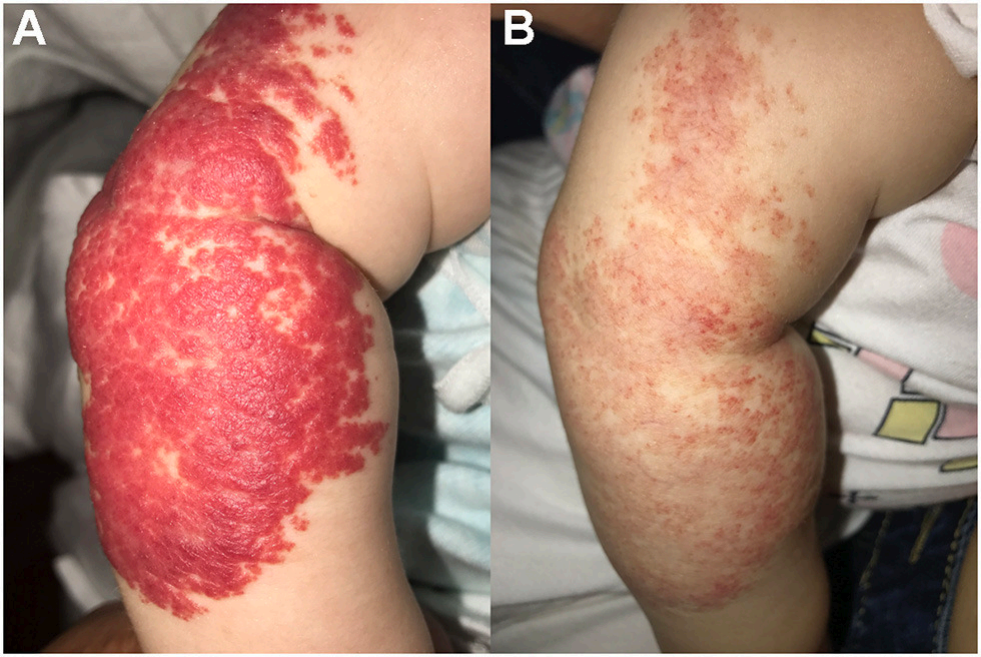
Therapeutic response of superficial IHs in the arm treated with topical timolol maleate 0.5% hydrogel. **(A)** Before starting topical timolol therapy; **(B)** After 6 months of topical timolol treatment. Significant discoloration and regression were observed. Written informed consent has been obtained from the parent of the child, and permission was granted for publication of these images.

Both propranolol and timolol achieved a satisfactory outcome in treating superficial IHs, and no significant differences in VAS improvement were observed in the present study (*P* = 0.20). As shown in Table 3, the average VAS improvement after oral propranolol treatment was 71.2, with 116 patients achieving excellent response (as shown in Figure 1), 199 patients achieving good response, 36 patients achieving fair response and 11 patients achieving poor response. The average VAS improvement after topical timolol treatment was 77.2, with 136 patients achieving excellent response (as shown in Figure 2), 170 patients achieving good response, 43 patients achieving fair response and 13 patients achieving poor response.

**Table 3:** Therapeutic evaluation of propranolol and timolol treatment.

**Efficacy**	**Excellent**	**Good**	**Fair**	**Poor**	***P*-value**
Propranolol	116	199	36	11	0.20
Timolol	136	170	43	13	

### Evaluation of Drug Adverse Events

As shown in Table 4, there was a remarkable difference in the incidence of systemic adverse events between the two groups. No systemic AEs were noted during topical timolol treatment, compared with 14 patients experiencing systemic AEs during oral propranolol treatment. Meanwhile, mild local side effects were observed in 12 patients treated with topical timolol, including local pruritus and skin blemishes.

**Table 4:** Evaluation of drug adverse events in patients treated with propranolol and timolol.

**Groups**	**Propranolol group**	**Timolol group**	***P*-value**
	**(*n* = 362)**	**(*n* = 362)**	
Local AEs	0	12	<0.001
Systemic AEs	14	0	<0.001

### Predictors for Clinical Responses

Predictors for clinical responses were listed in Table 5. Clinical response was not associated with gender, duration of treatment, lesion location, lesion size, gestational age, and progesterone use during pregnancy. The only predictor for clinical responses was age at treatment initiation for both groups, which indicated that better therapeutic effects were achieved in patients with younger age (<6 months old) at treatment initiation.

**Table 5:** Predictors for clinical response.

**Variables**	***P*-value**	***P*-value**
	**(Propranolol group)**	**(Timolol group)**
Gender	0.53	0.32
Age at treatment initiation	0.02^*^	0.04^*^
Duration of treatment	0.19	0.52
Lesion location	0.29	0.27
Lesion size	0.53	0.24
Gestational age	0.37	0.43
Progesterone use during pregnancy	0.28	0.31

* *p < 0.05 was considered as significant*.

### Therapeutic Effects for Large Hemangiomas

Large hemangiomas with an area larger than 5 cm^2^ are one of the most troubling problems. Therefore, we evaluated the therapeutic effects for large hemangiomas treated by propranolol or timolol. Both propranolol and timolol achieved a satisfactory outcome in treating large superficial IHs, and no significant differences in therapeutic effects were observed in the present study (*P* = 0.52). As shown in Table 6, 42 patients treated by oral propranolol achieved excellent response, with 65 patients achieving good response, 15 patients achieving fair response and 5 patients achieving poor response. In contrast, 47 patients treated by topical timolol achieved excellent response, with 50 patients achieving good response, 10 patients achieving fair response and 4 patients achieving poor response.

**Table 6:** Therapeutic evaluation for large lesions (tumor size ≧ 5 cm^2^).

**Efficacy**	**Excellent**	**Good**	**Fair**	**Poor**	***P*-value**
Propranolol	42	65	15	5	0.52
Timolol	47	50	10	4	

## Discussion

The treatment pattern of IHs has changed dramatically since the successful use of propranolol. A series of clinical studies including retrospective study, prospective study and meta analysis have been implemented to prove the efficacy of propranolol in treating IHs (15–17). However, the safety of propranolol therapy in IH hsa been controversial because of the systemic AEs induced by oral administration. Although, common systemic side effects including bradycardia, bronchospasm, hypotension, hypoglycemia, electrolyte disturbances and diarrhea, are usually self-limiting without special intervention (18), concerns regarding the potential effects of propranolol on neurocognitive ability have been raised recently. It is widely known that the lipophilic nature of propranolol could favor in penetrating the blood-brain barrier, but whether oral propranolol would affect the central nervous system in a long-term period is still unclear (19). Consequently, clinicians attempted to use topical β-blockers for treating IHs in order to minimize the side effects induced by oral administration. As for superficial lesions, topical medication could achieve local drug distribution and reduce the release of the drug into blood circulation. Although topical beta blockers have achieved acceptable effects for treating superficial IHs, there is still no consensus on the selection of oral propranolol or topical timolol for treating superficial IHs. Herein, we conducted a comparative cohort study to evaluate the outcomes of superficial IHs treated with either topical timolol or oral propranolol, and possible AEs. Our results based on large samples demonstrated that both oral propranolol and topical timolol are effective for treating superficial IHs, and there were no significant differences in efficacy between the two treatment modalities. Moreover, fewer systemic AEs were observed in patients receiving topical timolol than those receiving oral propranolol. This study provided supportive evidence of choosing topical timolol as the first-line therapy for superficial IHs.

Propranolol, as a non-selective β-blocker, could suppress the growth of IHs through inducing vasoconstriction, angiogenesis inhibition, and apoptosis induction (20). Recent studies have demonstrated that oral propranolol could achieve a satisfactory therapeutic response at a dosage of 2–3 mg/kg per day (1). Moreover, propranolol was proved to be a good choice for the treatment of obstructive, alarming and ulcerated lesions (1). In the present study, we applied propranolol at a dosage of 2 mg/kg per day, with an effective response rate of 97%, which is consistent with the results (96–98%) by Léauté-Labrèze et al. (1). These results further supported the good effects of propranolol for treating IHs.

As for topical drug therapy, diverse formulations of timolol, including timolol 0.1% gel, timolol 0.25% gel forming solution, timolol 0.5% eye drop, timolol 0.5% gel forming solution, and timolol 0.5% gel, have been attempted for treatment of superficial IHs (21). In our previous study, we applied topical timolol maleate 0.5% hydrogel for treating superficial IHs, and discovered that topical timolol could achieve satisfactory clinical responses with mild side effects (12). Propranolol and timolol are both β-blockers, which may regulate the growth of IHs in a similar way. However, few studies were conducted to compare the therapeutic effects of topical timolol and oral propranolol. According to recent review focus on the interventions for hemangiomas of the skin, only one study including 26 participants reported that no significant difference in haemangioma size after treated by oral propranolol or topical timolol was observed (22, 23). Consistent with this study, our results showed no significant differences in efficacy between the two treatment modalities, and both treatments can be adopted for superficial IHs.

To reduce systemic side effects induced by oral propranolol is one major reason for applying topical timolol as an alternative of treating IHs, but few clinical studies investigated the improvement of treatment safety by comparing the outcomes of patients treated with either oral propranolol or topical timolol. This study showed significant differences in the occurrence of AEs between the two groups. Fewer systemic AEs were observed in patients receiving topical timolol than those receiving oral propranolol. Of the 362 children in the propranolol group, 14 patients had systemic adverse reactions, including 10 with sleep disorders, 5 with diarrhea, 6 with loss of appetite, 4 with transient acromegaly, and 2 with bronchial spasm. Compared with the propranolol group, 362 patients in the timolol group had no systemic adverse drug reactions, and only 12 patients had local pruritus and skin blemishes. These findings indicated that the occurrence of systemic AEs for patients treated with oral propranolol was significantly higher than that for patients treated with topical timolol (*P* < 0.05). Therefore, we recommend topical timolol instead of oral propranolol as the first-line therapy for superficial IHs because of its good efficacy and improved safety.

As shown in the results, age at treatment initiation was closely associated with therapeutic efficacy in both groups, with a higher regression rate for patients younger than 6 months old treated with either topical timolol or oral propranolol. These results were consistent with previous studies, which disclosed that better regression rate of IH lesions could be achieved in patients younger than 6 months old (10, 24). We hypothesized that this phenomenon was due to the characteristic growth behavior of IHs. A rapid proliferation and followed regression is a distinct feature of IH. Rapid growth of IH lesions is usually observed during 5–8 weeks, and about 80% of their absolute growth is completed by the age of 3 months (25, 26). It is widely accepted that rapid proliferation and followed regression of IH lesions are closely associated with the crucial role of beta adrenergic receptor in regulating the growth of IHs (27). β-blockers including propranolol and timolol could elicit inhibitory effects mainly via regulating cell proliferation, angiogensis and apoptosis through beta adrenergic receptor signaling pathway. Moreover, the varied expressions of beta adrenergic receptor in proliferating and involuted lesions are in accordance with characteristic growing pattern of IHs (28). Consequently, we hypothesized that worse therapeutic effects at elder ages of treatment initiation were partially owing to decreased tendency of adrenergic receptor expression.

It is widely accepted that dysregulated differentiation of embryonic cells contributed to the progression of IHs, which is comprised of proliferative hemangioma endothelial cells forming the vessels and immature hemangioma pericytes circumscribing the vessels (29). According to recent studies focusing on the potential mechanisms of different anti-hemangioma drugs, propranolol and other beta-blockers mainly exert their effects via targeting hemangioma endothelial cells and hemangioma pericytes (30). Bischoff et al. proposed that propranolol could suppress the development of hemangiomas through increasing the contractility of hemangioma pericytes (31), and several other studies reported that propranolol could inhibit the growth of hemangiomas through modulating cellular functions of hemangioma endothelial cells (32–34). Although no significant differences in the therapeutic efficacy of propranolol group and timolol group in our study were observed, we hypothesized that systemic propranolol and local timolol might individually exerted their effects partly via targeting different cells. It is possible that systemic propranolol was dissolved in the vessels and firstly affected cellular physiology of hemangioma endothelial cells across the vessels, while topical timolol permeated through the skin and firstly interacted on hemangioma pericytes circumscribing the vessels. As a result, systemic propranolol might mainly target endothelial cells initially, and topical timolol might mainly target pericytes initially. However, few evidences provide support for our hypothesis. Therefore, more studies are needed to compare the potential mechanisms of local and systemic beta-blockers on treating hemangiomas.

## Conclusion

In the present study, we discovered that topical timolol is at least as effective as oral propranolol for the treatment of superficial IHs, and poses less risk of inducing systemic adverse events. Therefore, we recommend topical timolol as the first-line therapy for superficial IHs.

## Author Contributions

Conception/design: HW, XW, and JZ. Provision of study material and patients: HW. Collection and assembly of data: LZ, JZ, CL, and YW. Data analysis and interpretation, manuscript writing, final approval of manuscript: all authors.

### Conflict of Interest Statement

The authors declare that the research was conducted in the absence of any commercial or financial relationships that could be construed as a potential conflict of interest.
